# Cost-effectiveness of diabetic retinopathy screening programs using telemedicine: a systematic review

**DOI:** 10.1186/s12962-020-00211-1

**Published:** 2020-04-06

**Authors:** Daniel Avidor, Anat Loewenstein, Michael Waisbourd, Amir Nutman

**Affiliations:** 1 Sackler Faculty of Medicine, Tel-Aviv University (TAU), Tel-Aviv, Israel; 2 Ophthalmology Department, Tel-Aviv Sourasky Medical Center, Tel-Aviv, Israel; 3 Glaucoma Research Center, Tel-Aviv Sourasky Medical Center, Tel-Aviv, Israel; 4 Tel-Aviv Sourasky Medical Center and National Center for Infection Control, Tel-Aviv, Israel

**Keywords:** Diabetic retinopathy, Telemedicine screening, Teleophthalmology, Economic evaluation, Cost-effectiveness

## Abstract

**Background:**

Diabetic retinopathy (DR) is a significant global public health and economic burden. DR accounts for approximately 15–17% of all cases of total blindness in the USA and Europe. Telemedicine is a new intervention for DR screening, however, there is not enough evidence to support its cost-effectiveness. The aim of this study is to review the most recent published literature on economic evaluations of telemedicine in DR screening and summarize the evidence on the cost-effectiveness of this technology.

**Methods:**

A systematic search of PubMed, Embase and Google Scholar for relevant articles published between January 2010 and January 2020. Studies were included if they met the following criteria: (1) recruited subjects with either type 1, type 2 diabetes (2) evaluated telemedicine technology (3) patients underwent primary screening for DR (4) compared a telemedicine-based intervention with standard care (5) performed an economic evaluation or provided sufficient data for evaluating the cost-effectiveness of the technology used.

**Results:**

Of 2238 articles screened, seven studies were included. Four of the studies were conducted in developed countries: The United States, Singapore and two studies in Canada. Three studies were conducted in developing countries: India, Brazil and South Africa. The patient populations in all studies were diabetic patients over the age of 18, previously not screened for DR. All seven studies used a telemedicine program which included capturing a retinal image and subsequently transmitting it to an ocular imaging center to assess the severity of DR. All studies compared telemedicine to a standard screening method for DR, including the option of no screening as standard of care. Although telemedicine requires initial and maintenance costs, it has the potential to provide significant cost savings by increasing patients’ working ability, increasing independent living ability, increasing quality of life and reducing travel costs.

**Conclusions:**

Diabetic retinopathy telemedicine technology has the potential to provide significant cost savings, especially in low-income populations and rural patients with high transportation costs.

## Background

Diabetes mellitus (DM) incidence and prevalence is on the rise in recent years, causing significant morbidity and mortality [1]. In 2014, 422 million patients worldwide were diagnosed with DM, this number is expected to rise to 592 million by 2035, compared to 108 million in 1980. In the European region, there are about 60 million people with DM comprising 10.3% of men and 9.6% of women aged 25 years and over [2]. In the United States, there are 30.2 million people aged 18 and over with DM, which represent 12.2% of the entire population; 11.7% of the women and 12.7% of the men [3].

Diabetic retinopathy (DR) is a common microvascular complication of DM, which causes irreversible damage to the retina. The World Health Organization (WHO) has estimated that DR accounts for approximately 15–17% of all cases of total blindness in the USA and Europe [4]; it is the leading cause of blindness amongst the working-age population in developed countries [5]. More than 60% of individuals with type 2 DM and virtually all patients with type 1 DM develop DR within the first 20 years of diagnosis of the disease [6]. With the increasing prevalence of DM, the number of individuals with DR is also likely to rise. Globally, the number of people with DR is expected to grow from 126.6 million in 2010 to 191.0 million by 2030, and some studies estimate that the number of patients with vision-threatening DR will increase from 37.3 to 56.3 million [7]. These disturbing numbers make DR a significant global public health and economic issue.

Multiple studies and clinical trials have reported the benefit of early detection and timely treatment in reducing the risk of vision loss from DR [8] and decreasing the global burden of blindness [9]. Active screening for DR is important because most patients who develop DR have no symptoms until the very late stages, and by then it is often too late for effective treatment. Although there are several ways to detect DR, the gold standard is a dilated fundus examination using an indirect ophthalmoscope or a slit lamp bio-microscope by an ophthalmologist. The American Academy of Ophthalmology recommends annual DR examinations for patients with type 1 and type 2 DM, however, in practice only 50% to 65% of patients receive this recommended screening [10].

Multiple patient barriers to DR screening exist, including poor access to care, lack of time, high out-of-pocket expenses, insufficient patient knowledge and awareness of DR, and lack of care coordination, especially among low-income populations, and ethnical minorities [11, 12]. These barriers are further magnified among developing countries [11, 13].

Telemedicine is the exchange of medical data using electronic technology that allows a patient’s medical case to be evaluated and monitored by a remotely located physician [11]. Telemedicine screening programs to assess DR disease through remote retinal imaging can effectively diagnose DR and recommend proper care interventions [14]. There is a variety of retinal imaging technologies, such as digital imaging systems, hand-held fundus cameras, non-mydriatic cameras and the use of smartphones as a fundus camera [15]. The goal of telemedicine—teleophthalmology programs in DR is to increase the number of patients screened and monitor those at risk for progression [12]. Teleophthalmology screening programs have the potential to increase access to care in remote areas, save the patient time and travel costs, and to identify those who have the immediate need for retinal evaluation versus those who do not [14, 16].

Teleophthalmology can produce the same clinical outcomes as the traditional face to face examination [17]. Tan et al. [18] published a systematic review of teleophthalmology diagnostic accuracy compared with face to face consultation and found that teleophthalmology was considered superior to face to face consultation in one study and comparable in six studies. Surendran et al. [19] demonstrated that overall, the published medical literature agrees that teleophthalmology programs are accurate and safe alternatives to the traditional DR screening.

Although studies have examined the clinical benefits of telemedicine [16], the use of teleophthalmology is currently in its infancy and has yet to gain widespread acceptance, and only a few studies have assessed the economic benefits. The purpose of this systematic review is to summarize the data on economic evaluation and cost-effectiveness of DR screening in type 1 and 2 diabetes patients using teleophthalmology technology as compared to standard care.

## Methods

The systematic review protocol was not registered in any database. A literature search was performed using PubMed, Embase and Google Scholar search engines for relevant articles published between January 2010 and January 2020. We used a combination of medical subject headings and text terms to generate four subsets of citations: one for diabetic retinopathy, the second for telemedicine, based on a search using the terms ‘telemedicine’, ‘telehealth’, ‘teleophthalmology’ and ‘teleretinal’, the third used the term ‘screening’ and the fourth for economic analysis based on the terms ‘cost-effectiveness’, ‘cost-utility’, ‘cost–benefit’, ‘incremental cost-effectiveness ratio’, ‘economic analysis’ and ‘quality of life years’. The terms were combined to generate a subset of citations relevant to the research question. Also, we examined the reference lists in original and review articles to identify additional studies that were not captured by the electronic searches. Articles in languages other than English and narrative reviews were excluded. We included studies if they met the following criteria: (1) recruited subjects with either type 1 or type 2 DM (2) evaluated DR telemedicine technology (3) patients underwent primary screening for DR (4) compared a telemedicine-based intervention with standard care (5) performed an economic evaluation or provided sufficient data for evaluating the cost-effectiveness of the technology used. Studies that included patients with known DR or patients with other co-morbid eye disease were excluded, as well as studies with no description of subjects. All identified studies were exported into the citation software package ‘Mendeley’ and duplicates were removed. We included only published data, which may lead to an inherent problem of publication bias.

## Results

**Fig. 1:**
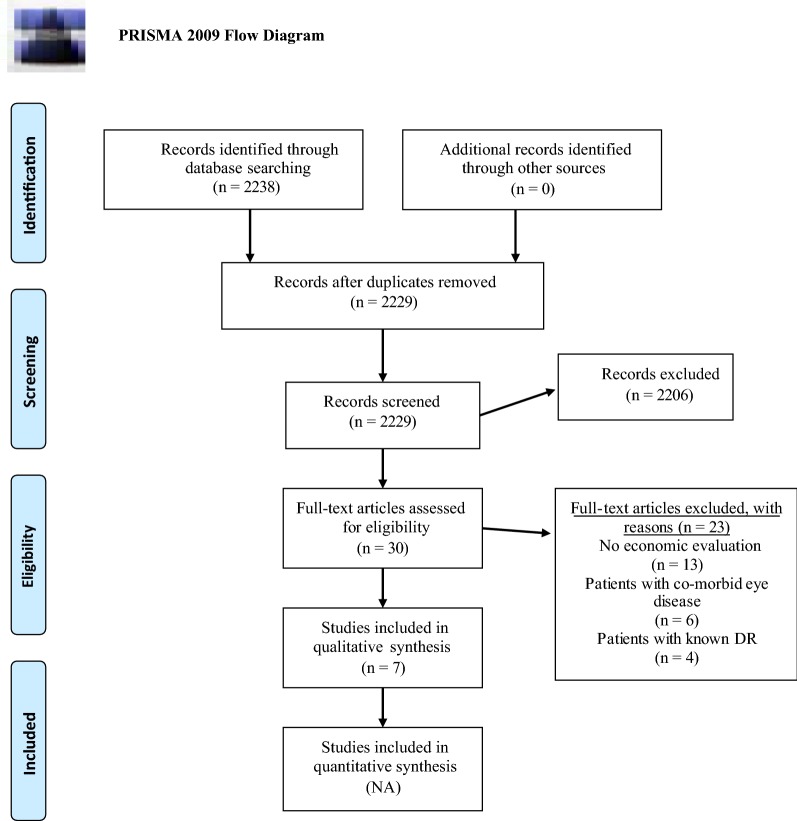
Flow diagram of study selection. Depicts the flow of the studies included in this article. Maps out the number of studies identified, included and excluded, and the reasons for exclusions

Of 2238 articles screened, 30 full-text articles were assessed for eligibility criteria, and seven studies were included in this review (Fig. 1).

Four of the studies were conducted in developed countries: The United States, Singapore and two studies in Canada. Three studies were conducted in developing countries: India, South Africa and Brazil (Table 1). All studies reported the economic analyses using local currency, except for one. To ease the comparison between studies, we converted local currency to United States dollars (USD) based on the exchange rate at the time of writing this manuscript. The patient populations in all studies were diabetic patients over the age of 18, without known DR.

**Table 1:** Presents in short the difference between articles

First Author, Year, Country	Population size	DM type	Comparator	Screening modality	Outcome	Results	*Quality of the evidence* GRADE^a^ and CEBM^b^
Rachapelle, 2013, India	1000	Not specified	No screening	Mobile van with an a-built-in ophthalmic unit in which an optometrist took retinal images that were transferred by satellite to the base hospital, where they were reviewed by an ophthalmologist and graded using the international DR classification system	ICER^c^	From the health provider perspective: Screening every 2 years—$2435 per QALY^d^ gained (within cost-effective range); Annual screening—$4029 per QALY gained (outside cost-effective range). Annual screening—not in the cost-effective range From the societal perspective: Screening every 5 years—$3134 per QALY gained (within cost-effective range). Screening every 2 years—$3669 per QALY gained (outside cost-effective range)	*Moderate* Due to risk of bias CEBM: *3b* Individual case–control study
Kirkizlar, 2013, US	900	Type 1 and type 2 DM	Eye care professional performing a conventional retinal examination	Digital images were taken by a technician and sent electronically to a central location for reading by a retinal specialist or certified reader	Cost per QALY^d^	$46,449 per QALY for screening 3500 patients (decreasing as patient pool size increasing) Cost/QALY for patients over 50 years—$7228 (increasing as patient age increasing)	*Low* Due to the risk of bias and Imprecision CEBM: *3b* Individual case–control study
Nguyen, 2016, Singapore	Hypothetical cohort of patients	Type 2 DM	Primary care physician	Retinal images were transmitted to an ocular imaging center. Trained graders assessed the severity of DR and sent the results back to the primary physician (within a 1-h turnaround time)	Cost per QALY^d^	The telemedicine-based DR screening generates cost savings of $127 saving per patient with similar QALY	*Low* Due to the risk of bias and indirectness of evidence CEBM: *3b* Individual case–control study
Khan, 2013, South Africa	14,541	Type 2 DM	No screening	Retinal photographs were taken by mobile camera transport in a vehicle and read by a medical officer with ophthalmic experience	ICER and costs	$1206 per blindness case averted Potential cost-savings—$19,310,344 per year	*Low* Due to the risk of bias and Imprecision CEBM: *3b* Individual case–control study
Kanjee, 2017, Canada	4676	Type 2 DM	The existing service, the retinal specialist provides in-clinic screening	Retinal images transmitted to a centralized reading facility and graded by a retinal specialist	Costs	The telemedicine program produced savings of $752 per examination performed	*Low* Due to the risk of bias and Imprecision CEBM: *3b* Individual case–control study
Ben, 2020, Brazil			(1) Opportunistic Ophthalmologist referral to secondary care individuals who seek medical attention at primary care. (2) Systematic ophthalmology referral for all individuals with diabetes	Systematic teleophthalmology-based. Retinal images were taken from individuals and were sent to a remote ophthalmology center for evaluation	Costs and ICER and QALY	The systematic teleophthalmology based screening was more effective although more expensive, with an additional cost of $209 and incremental QALY of 0.042; thus the ICER of this intervention was $4976/QALY	*Moderate* Due to the risk of bias CEBM: *3b* Individual case–control study
Stanimirovic, 2019, Canada (Toronto)	566	Type 1 and type 2 DM	Fundus examination by optometrist or ophthalmologist	Retinal images were taken and uploaded to a server and graded for the level of DR by a retina specialist	Costs and ICER	The teleretinal screening program correctly diagnosed more patients (496 vs. 247) and was cost-saving ($82.4 vs. $237.8)	*Moderate* Due to the risk of bias CEBM: *3b* Individual case–control study

^a^The Grading of Recommendations Assessment, Development and Evaluation working group approach to grading quality (or certainty) of evidence and strength of recommendations

^b^The Centre for Evidence-Based Medicine hierarchy of the quality of medical research evidence, named the levels of evidence

^c^Incremental cost-effectiveness ratio: the difference in cost between two interventions, divided by the difference in their effect. It represents the average incremental cost associated with 1 additional unit of the measure of effect

^d^*Quality Adjusted Life Year*. A generic measure of disease burden including both the quality and the quantity of life lived

All studies included used a telemedicine program which included capturing a retinal image and subsequently transmitting it to an ocular imaging center to assess the severity of DR. All studies compared telemedicine to a standard screening method for DR, including the option of no screening as the standard of care.

Kirkizlar et al. screened a total of 900 patients aged 18–99 years with type 1 and type 2 DM. The participants were from the Veterans Health Administration department in the USA and from different ethnic groups [20]. They used the economic model of Chapman et al. [21] to estimate the cost-utility of DR screening using telemedicine vs. conventional DR screening examination and considered cost-effectiveness if the cost per QALY of the program is less than an established monetary threshold of $50,000. They demonstrated that population size and age determined the cost-effectiveness of the program. A telemedicine screening program is cost-effective if the screened population size is over 3500 people. For a population of 3500 patients included in a screening program, the average costs are $46,449 per quality-adjusted life years (QALY) vs. $20,392 per QALY for a population size of 9000. However, telemedicine screening for patients under the age of 50 years or older than 80 years was not cost-effective (Additional file 1: Table S1).

Rachapelle et al. [22] used a Markov model to estimate the cost-utility of a telemedicine screening program with different screening intervals in comparison with no screening, which was the current standard of care in India. The study was based on a hypothetical cohort of 1000 rural diabetic patients aged 40 years. The costs and outcomes were estimated for a period of 25 years. They estimated the costs per person from a healthcare provider perspective and societal perspective (the sum of the health provider and household costs). Utility was defined using the time trade-off method by interviews with diabetic patients; the maximum number of years the patient was willing to trade for perfect vision. The incremental cost-effectiveness ratio (ICER) threshold used to determine cost-effectiveness was < $3183 per QALY gained, using WHO recommendations. Their cost-utility model suggested that from a health provider’s perspective, the cost of telemedicine screening once every 2 years was $2435 per QALY gained, which is cost-effective compared to no DR screening for rural diabetic patients. Annual screening fell outside of the cost-effective range, $4029 per QALY gained, however, with 85% of the additional costs attributable to hospital fees. From a societal perspective, the cost of screening every 5 years was $3134 per QALY gained, which was considered cost-effective. However, the cost of screening every 2 years at $3669 per QALY gained, was not considered cost-effective.

Nguyen et al. [23] used a Markov model to estimate the cost-effectiveness from the health system and societal (medical and non-medical costs) perspectives. They used the UK National Institute for Health and Care Excellence (NICE)’s ICER threshold of £30,000 per QALY to determine cost-effectiveness. The study population consisted of a hypothetical cohort of patients with type 2 DM. The mean age was 55 years, which reflected the actual age distribution of the Singaporean population of patients with type 2 DM. They reported that over a lifetime telemedicine was cost-saving, from both the health system and societal perspectives, compared with the current practice, a family physician eye examination, with similar QALYs across the two programs. The main savings were from the societal perspective; every patient with DM who used telemedicine would generate cost savings of approximately $127 USD.

Khan et al. [24] analyzed the cost-effectiveness of a telemedicine screening program in South Africa. Patients were seen in three community health centers and all of them were from low social-economic backgrounds. All patients had type 2 DM. The analysis included only direct medical costs, as well as transportation costs. The comparator was defined as the current practice, where people with DM were not screened for DR. The outcome measure was cost per one blindness case averted. They calculated that the cost for telemedicine screening was $22 per person and the ICER was $1206 per blindness case averted; less than the threshold used which was the annual disability grant in South Africa ($1393). A telemedicine DR screening program would be cost-effective even if just 65% of people with DM were screened. The main limitation of this study was that it took place in Cape Town, which is different from other parts of South Africa as; DR prevalence and the stage at which patients present to health care are lower than other parts of South Africa. Additionally, costs will initially increase in areas that have a significantly high prevalence of DM, but savings will occur in the long term.

Kanjee et al. [25] estimated the costs of a telemedicine program in a Manitoban cohort. They performed a retrospective chart analysis of 4676 patients from the Manitoba Retinal Screening Vision Program. Patients were included if they were 18 years or older and had a diagnosis of type 2 DM. They demonstrated a lower cost for telemedicine compared with conventional in-clinic screening. On average, the telemedicine program produced savings of $752 per examination performed, compared to in-clinic screening.

Ben et al. [26] performed a model-based economic evaluation to compare three DR screening practices in Brazil. The population included were patients with type 2 DM aged 40 years and without known DR. Analyses were performed from the Healthcare System perspective. The three DR screening strategies were: (1) the common practice, opportunistic ophthalmology referral, i.e. ophthalmologist referral for individuals who seek medical attention at primary care (ophthalmologist referral is covered by the public primary care program in Brazil). (2) Systematic ophthalmologist referral, i.e. ophthalmologist referral for all individuals with type 2 DM (3) systematic teleophthalmology referral where retinal images were sent to a remote ophthalmology center for evaluation. The ICER threshold was determined based on the GDP per capita for Brazil. Interventions with an ICER less than $10,382/QALY were considered cost effective. The systematic teleophthalmology based screening was more effective although more expensive: an additional cost of $209 and incremental QALY of 0.042; thus the ICER of this intervention was $4976/QALY, which is under the ICER threshold ($14,953/QALY).

Stanimirovic et al. [27] assessed the cost-effectiveness of a pilot Toronto teleophthalmology screening program using a decision tree model. Images were taken by the primary care provider and analyzed by a retina specialist. A total of 566 patients aged > 20 years, with both type 1 and 2 DM were screened. The economic analysis was conducted from a health care perspective and the cost-effectiveness of teleophthalmology screening was assessed as cost per case detected (true-positive) and cost per case correctly diagnosed (true-positive and true-negative). Compared to conventional screening by a primary care eye specialist, the teleophthalmology screening program correctly diagnosed more patients (496 vs. 247) and was cost-saving ($82.4 vs. $237.8). Thus, the teleophthalmology program was the dominant strategy (ICER < 0).

## Discussion

Early detection, accurate diagnosis, and timely treatment of DR have long been established as a means to significantly reduce vision loss from DM and improve public health [15]. Multiple professional organizations, including the American Academy of Ophthalmology, recommend annual retinal examinations for patients with DM [10]. Despite these recommendations and the known fact that screening for DR is a cost-effective method to reduce blindness [24], many DM patients do not receive recommended screening. In the United States and other developed countries, only half of the diagnosed population with DM is screened annually for DR [28], therefore diabetes-related vision loss remains the major cause of blindness in western populations [15]. We can only assume that in developing countries the proportion of screened patients is likely even lower. The reasons for poor compliance are several, including lack of patient education, lack of access to care, and geographic limitations [12]. Gibson et al. [29] demonstrated that 73% of patients with DR unaware of their condition.

Telemedicine can overcome geographical, financial, and socioeconomic barriers. Blomdahl et al. [30] reported that teleophthalmology is particularly useful when the distance to an ophthalmologist is an obstacle to diagnosis and treatment. Teleophthalmology can expand standards of eye care delivery by extending access to care, offering alternative methods for receiving appropriate care, and integrating DM eye care into the patient’s total healthcare. A high satisfaction level and acceptance from patients is reported in the majority of the studies because of increased accessibility and reduced traveling cost and time [17].

In this study, we evaluated the cost-effectiveness of teleophthalmology programs reported in a variety of settings, including the United States, Canada, Singapore, India, Brazil, and South Africa. In all studies teleophthalmology was cost-effective based on the results of the economic evaluations. Kirkizlar et al. [20] demonstrated that a teleophthalmology screening program is cost-effective for a population size of over 3500 patients aged 50–80 years in the US. Rachapelle et al. [22] reported that telemedicine screening programs are cost-effective compared with no DR screening for Indian rural diabetic patients. Khan et al. [24] demonstrated that teleophthalmology is a cost-effective screening tool for DR in a South African population, even if merely 65% of people with DM are screened. Nguyen et al. [23] reported that every patient with DM who uses the telemedicine program generates cost savings of $127 over a lifetime. Currently, there are 170,000 DM patients in Singapore, thus the expected total savings if a teleophthalmology screening program would be implemented is estimated to be $21.6 million (170,000 × $127). Kanjee et al. [25] demonstrated a lower cost to the telemedicine program compared to the conventional in-clinic screening in a Canadian cohort. Ben et al. [26] presented that systematic teleophthalmology based screening is cost-effective compared to opportunistic ophthalmology-referral based screening in Brazil. Stanimirovic et al. [27] found that teleophthalmology screening dominated the standard of care in a Canadian pilot study (ICER < 0).

The findings of this systematic review should be interpreted with caution due to the lack of data from randomized controlled trials (RCTs) and high risk of bias in the available data from observational studies. Another limitation is that DR screening was performed in each study by different health care professionals such as optometrists, general physicians, and clinical photographers and using different modalities, which makes it difficult to synthesize results.

Although telemedicine requires initial and maintenance costs, we conclude from this review that this intervention has the potential to provide significant cost savings via accurate DR diagnosis and treatment, thus increasing patients working ability, increasing independent living ability, increasing quality of life and reducing travel costs, and are cost-effective from both the healthcare and a societal perspectives. These programs are even more cost-effective in low-income populations and rural diabetic patients with high transportation costs [31–33]. With advances in technology which include better and faster telecommunication, cloud storage, miniaturization of equipment (smartphones with digital cameras) and automation of retinal image analysis, teleophthalmology screening programs can be optimized. This optimization includes improving productivity, safety standards, quality assurance, and sustainability, thus improving patient care and long-term outcomes [11, 34]. As the costs of equipment are constantly reduced, teleophthalmology services costs are reduced, thus improving cost-effectiveness.

## Conclusion

Teleophthalmology is an emerging technology with the potential to improve accessibility to DR screening programs and positively influence patient care on a large scale. This intervention has the potential to improve compliance with DR screening and reduce the incidence of vision-threatening complications of diabetes, thus increasing patients’ working ability, independent living ability and quality of life, as well as reduce costs such as traveling costs and physician time. Although our review identified only a few economic evaluations of screening DR with telemedicine programs to date, these studies demonstrated cost-effectiveness. We propose that more robust RCTs will be conducted to evaluate the application of teleophthalmology to monitor DR and other eye conditions. In the future, with the aid of automated systems for grading DR using artificial intelligence (AI), this technology has the potential to be even more accurate, save physician specialist time, and improve the cost-effectiveness of the DR screening service.

## Supplementary information

**Additional file 1: Table S1.** Table of full text articles excluded with reasons.

## Data Availability

All data generated or analyzed during this study are included in this published article.

## Abbreviations

DR: Diabetic retinopathy
DM: Diabetes mellitus
ICER: Incremental cost-effectiveness ratio
QALY: Quality-adjusted life years
