# Supplementary material for: Cost-effectiveness of diabetic retinopathy screening programs using telemedicine: a systematic review
Source: Cost Eff Resour Alloc. 2020 Apr 6;18:16. doi: 10.1186/s12962-020-00211-1 (PMC7137317; doi:10.1186/s12962-020-00211-1)
Supplement: Supplementary file 1 — Additional file 1: Table S1. Table of full text articles excluded with reasons. [file 12962_2020_211_MOESM1_ESM.docx]

**Additional file 1: Table S1**

| **First Author, Year** | **Population of interest** | **Intervention** | **Control** | **Outcome** | **Reason for exclusion** |
| --- | --- | --- | --- | --- | --- |
| Mansberger, 2015  (1) | Patients with diabetes aged >18 years. | Retinal imaging during a regular primary care clinic visit. | Eye examination by eye care professional. | Percentage of patients who received DR; percentage of telemedicine examinations requiring referral to an eye care professional; percentage of eyes that had higher, lower, or the same level of DR. | No economic evaluation |
| Mansberger, 2013  (2) | Patients with diabetes aged >18 years. | Retinal imaging during a regular primary care clinic visit. | Eye examination by eye care professional. | Proportion of DR screening examinations; prevalence and stage of DR; risk factors for DR. | No economic evaluation |
| Olayiwola, 2011 (3) | Patients with diabetes. | Digital retinal images by numerous staff. | None. | Percentage of patients who set a self-management goal; percentage of patients who aren’t ready to set SMG; percentage of patients who did not set an SMG during the visit. | No economic evaluation |
| Owsley, 2015 (4) | Patients with diabetes aged >18 years. | Ocular imaging by trained technicians. | None. | Percentage of other ocular findings; percentage of DR in minority groups; the percentage of patients with health insurance; percentage of participants with specific types of DR. | No economic evaluation |
| Zhijian, 2012 (5) | Patients with diabetes aged 28-77. | Digital Retinal Imaging. | Standard Ophthalmologic Evaluation. | Percentage of patients who have DR; patients screened positive with clinically significant disease. | Co-morbid eye disease |
| Rein, 2011 (6) | Patients with type 2 diabetes or retinal  microaneurysms aged 30-90 years. | Retinal Imaging in a primary care office. | Patient self-referral; annual eye evaluation; biennial eye evaluation | Costs and benefits of three screening scenarios to each other and to a counterfactual of self-referral. | Patients with known DR |
| Coronado, 2016 (7) | Patients with diabetes aged >15 years. | Retinal imaging in a pharmacy. | In-person examination. | Prevalence of any DR; the screening rate; volume increase of screening compliance. | Patients with known DR |
| Kurji, 2013 (8) | Patients from diabetic clinic. | Digital fundus images in a diabetic clinic. | None. | Patient preferences questionnaire. | No economic evaluation |
| Tufail, 2017 (9) | Patients from diabetic clinic. | Automated DR image assessment systems. | Human graders. | Screening performance; economic analysis estimated the cost per screening. | Patients with known DR |
| Garoon, 2018 (10) | Adult diabetic patients. | Retinal imaging. | None. | Costs and cost savings. | Co-morbid eye disease |
| Martínez Rubio, 2012 (11) | Adult diabetic patients. | Retinal imaging. | None. | Prevalence of DR; Percentage of mild-moderate proliferative DR; Percentage of non-proliferative DR, Percentage of proliferative DR; Percentage of DR. | No economic evaluation |
| Malerbi, 2015 (12) | Patients with type 1 DM. | Mydriatic 2 field retinography. | mydriatic BIO. | Clinical outcome was either observation or referral to the ophthalmologist (moderate or severe non-proliferative diabetic retinopathy, proliferative diabetic retinopathy, or apparently present diabetic macular edema) | No economic evaluation |
| Daskivich, 2017 (13) | Patients with diabetes aged >18 years. | Retinal imaging during a regular primary care clinic visit. | None. | Screening rates and screening wait rates. | No economic evaluation |
| Hussain, 2017 (14) | Diabetic patients. | Retinal imaging in a special equipped van. | None. | Prevalence of DR subtypes and odds ratios for development of DR and proliferative DR. | No economic evaluation |
| Verma, 2020 (15) | DR patients. | Retinal imaging. | None. | The frequency of a PPL distribution; frequencies of mild non-proliferative DR, moderate NPDR, severe NPDR and PDR. | Patients with known DR |
| Liu, 2019 (16) | Adult diabetic patients. | Retinal imaging via ultra-wide-field (UWF) imaging. | Conventional early  treatment DR Study. | Percentage of retinal neovascularization detection and PDR pathologies. | No economic evaluation |
| Walton, 2016 (17) | Patients with diabetes ages 18-98. | Intelligent Retinal Imaging System (IRIS). | Manual interpretation. | The IRIS algorithm positive predictive; the IRIS algorithm negative predictive value. | No economic evaluation |
| Perilli, 2016 (18) | Adult diabetic patients. | Retinal imaging. | None. | Different stages of DR, AMD and Glaucoma. | Co-morbid eye disease |
| Romero-Aroca, 2016 (19) | Adult diabetic patients. | Non-mydriatic fundus camera unit. | In-person examination. | Cost of visits, examinations and interventions carried out for each patient; classification of DR severity; cost of 2.5-year screening program. | Co-morbid eye disease |
| Phan, 2014 (20) | Adult diabetic patients. | Retinal imaging. | None. | Fiscal cost of teleretinal screening; costs of primary care clinic visit. | No economic evaluation |
| Brady, 2014 (21) | Adult diabetic patients. | Non-mydriatic fundus photography with remote grading. | None. | Direct medical costs; teleophthalmology costs. | Co-morbid eye disease |
| Fonda, 2020 (22) | Adult diabetic patients. | Retinal imaging. | Conventional examination. | Cost-effectiveness compared with a conventional examination; DR examination rate; DR and DME epidemiology. | Co-morbid eye disease |
| VanAltsine, 2012 (23) | Patients with type 2 DM. | Retinal imaging. | None. | Costs of transportation and DR grading | No economic evaluation |

**Bibliography**

1. Mansberger SL, Sheppler C, Barker G, Gardiner SK, Demirel S, Wooten K, et al. Long-term comparative effectiveness of telemedicine in providing diabetic retinopathy screening examinations: A randomized clinical trial. JAMA Ophthalmol. 2015 May 1;133(5):518–25.

2. Mansberger SL, Gleitsmann K, Gardiner S, Sheppler C, Demirel S, Wooten K, et al. Comparing the Effectiveness of Telemedicine and Traditional Surveillance in Providing Diabetic Retinopathy Screening Examinations: A Randomized Controlled Trial. Telemed e-Health [Internet]. 2013 Dec 1 [cited 2020 Mar 2];19(12):942–8. Available from: https://www.liebertpub.com/doi/10.1089/tmj.2012.0313

3. Olayiwola JN, Sobieraj DM, Kulowski K, Hilaire DS, Huang JJ. Improving diabetic retinopathy screening through a statewide telemedicine program at a large federally qualified health center. J Health Care Poor Underserved. 2011;22(3):804–16.

4. Owsley C, McGwin G, Lee DJ, Lam BL, Friedman DS, Gower EW, et al. Diabetes eye screening in urban settings serving minority populations: Detection of diabetic retinopathy and other ocular findings using telemedicine. JAMA Ophthalmol. 2015 Feb 1;133(2):174–81.

5. Li Z, Wu C, Olayiwola JN, Hilaire DS, Huang JJ. Telemedicine-based digital retinal imaging vs standard ophthalmologic evaluation for the assessment of diabetic retinopathy. Conn Med. 2012 Feb;76(2):85–90.

6. Rein DB, Wittenborn JS, Zhang X, Allaire BA, Song MS, Klein R, et al. The cost-effectiveness of three screening alternatives for people with diabetes with no or early diabetic retinopathy. Health Serv Res. 2011;46(5):1534–61.

7. Coronado AC, Zaric GS, Martin J, Malvankar-Mehta M, Si FF, Hodge WG. Diabetic retinopathy screening with pharmacy-based teleophthalmology in a semiurban setting: a cost-effectiveness analysis. C Open. 2016 Mar 8;4(1):E95–102.

8. Kurji K, Kiage D, Rudnisky CJ, Damji KF. Improving diabetic retinopathy screening in Africa: Patient satisfaction with teleophthalmology versus ophthalmologist-based screening. Middle East Afr J Ophthalmol. 2013 Jan;20(1):56–60.

9. Tufail A, Rudisill C, Egan C, Kapetanakis V V., Salas-Vega S, Owen CG, et al. Automated Diabetic Retinopathy Image Assessment Software: Diagnostic Accuracy and Cost-Effectiveness Compared with Human Graders. Ophthalmology. 2017 Mar 1;124(3):343–51.

10. Garoon RB, Lin W V, Young AK, Yeh AG, Chu YI, Weng CY. Cost Savings Analysis for a Diabetic Retinopathy Teleretinal Screening Program Using an Activity-Based Costing Approach. [cited 2020 Mar 10]; Available from: https://doi.org/10.1016/j.oret.2018.01.020

11. Martínez Rubio M, Moya Moya M, Bellot Bernabé A, Belmonte Martínez J. Diabetic retinopathy screening and teleophthalmology. Arch la Soc Española Oftalmol (English Ed. 2012 Dec 1;87(12):392–5.

12. Malerbi FK, Morales PH, Farah ME, Drummond KRG, Mattos TCL, Pinheiro AA, et al. Comparison between binocular indirect ophthalmoscopy and digital retinography for diabetic retinopathy screening: The multicenter Brazilian Type 1 Diabetes Study. Diabetol Metab Syndr [Internet]. 2015 Dec 21 [cited 2020 Mar 2];7(1):116. Available from: http://www.dmsjournal.com/content/7/1/116

13. Daskivich LP, Vasquez C, Martinez C, Tseng CH, Mangione CM. Implementation and evaluation of a large-scale teleretinal diabetic retinopathy screening program in the los angeles county department of health services. JAMA Intern Med. 2017 May 1;177(5):642–9.

14. Hussain N, Edraki M, Tahhan R, Sanalkumar N, Kenz S, Akasha NK, et al. Telemedicine for diabetic retinopathy screening using an ultra-widefield fundus camera. Clin Ophthalmol. 2017 Aug 14;11:1477–82.

15. Verma A, Alagorie AR, Ramasamy K, van Hemert J, Yadav N, Pappuru RR, et al. Distribution of peripheral lesions identified by mydriatic ultra-wide field fundus imaging in diabetic retinopathy. Graefe’s Arch Clin Exp Ophthalmol. 2020;

16. Liu TYA, Arevalo JF. Wide-field imaging in proliferative diabetic retinopathy. Vol. 5, International Journal of Retina and Vitreous. BioMed Central Ltd.; 2019.

17. Walton OB, Garoon RB, Weng CY, Gross J, Young AK, Camero KA, et al. Evaluation of automated teleretinal screening program for diabetic retinopathy. JAMA Ophthalmol. 2016;134(2):204–9.

18. Perilli R, Di Biagio R, Seller R, Ruotolo L, Granchelli C, Marisi V, et al. Teleretinography into diabetes integrated care: an Italian experience. Ann Ist Super Sanità. 2016;52(4):598–602.

19. Romero-Aroca P, De La Riva-Fernandez S, Valls-Mateu A, Sagarra-Alamo R, Moreno-Ribas A, Soler N, et al. Cost of diabetic retinopathy and macular oedema in a population, an eight year follow up. BMC Ophthalmol [Internet]. 2016 Aug 4 [cited 2020 Mar 9];16(1):136. Available from: http://bmcophthalmol.biomedcentral.com/articles/10.1186/s12886-016-0318-x

20. Phan ADT, Koczman JJ, Yung CW, Pernic AA, Doerr ED, Kaehr MM. Cost analysis of teleretinal screening for diabetic retinopathy in a county hospital population. Diabetes Care. 2014 Dec 1;37(12):e252–3.

21. Brady CJ, Villanti AC, Gupta OP, Graham MG, Sergott RC. Tele-ophthalmology screening for proliferative diabetic retinopathy in urban primary care offices: An economic analysis. Ophthalmic Surg Lasers Imaging Retin. 2014 Nov 1;45(6):556–61.

22. Fonda SJ, Bursell S-E, Lewis DG, Clary D, Shahon D, Horton MB. The Indian Health Service Primary Care-Based Teleophthalmology Program for Diabetic Eye Disease Surveillance and Management. Telemed e-Health [Internet]. 2020 Jan 31 [cited 2020 Mar 9];tmj.2019.0281. Available from: https://www.liebertpub.com/doi/10.1089/tmj.2019.0281

23. Cost-Effectiveness of Telemedicine Screening for Diabetic Retinopathy | IOVS | ARVO Journals [Internet]. [cited 2020 Mar 9]. Available from: https://iovs.arvojournals.org/article.aspx?articleid=2350814
